# An 8–18 GHz 90° Switched T-Type Phase Shifter

**DOI:** 10.3390/mi14081569

**Published:** 2023-08-07

**Authors:** Jialong Zeng, Yuxin Ren, Cheng Tan, Yang Yuan, Jiaxuan Li, Zhongjun Yu

**Affiliations:** 1 Aerospace Information Research Institute, Chinese Academy of Sciences, Beijing 100190, China; zengjialong19@mails.ucas.ac.cn (J.Z.); renyuxin211@mails.ucas.ac.cn (Y.R.); tancheng@aircas.ac.cn (C.T.); yuanyang19@mails.ucas.ac.cn (Y.Y.); lijiaxuan201@mails.ucas.ac.cn (J.L.); 2 School of Electronic, Electrical and Communication Engineering, University of Chinese Academy of Sciences, Beijing 101408, China

**Keywords:** T-type phase shifter, ultra wideband, filtering compensation network, relative phase shift

## Abstract

This paper proposes a novel 8–18 GHz 90° switched T-type phase shifter (TPS). In contrast to the conventional TPS, the proposed TPS adds a compensation capacitance to greatly enhance the phase shifting capacity. Moreover, the designed structure also integrates a filtering compensation network, which can effectively achieve a flat relative phase shift in a wide band. The proposed 90° TPS is fabricated using 0.15 μm GaAs pHEMT technology. The TPS achieves homogeneous phase shift at 8–18 GHz, with the measured phase error of less than ±1.5°. The insertion loss of the proposed phase shifter is 1.3–2.6 dB, and the chip size is merely 0.53 × 0.86 mm^2^. Thanks to these excellent performance characteristics, the designed phase shifter is well-suited for ultra-wideband wireless communication and radar systems.

## 1. Introduction

In recent years wireless communication and radar systems have developed rapidly, resulting in greater radar detection range and higher radar detection accuracy. These increasing demands call for high-power and wide-scanning radio frequency (RF) systems. In order to achieve these performances, the systems usually require thousands of channels to provide support. Therefore, the integration of the RF front-end has become the key to development. The emergence of monolithic microwave integrated circuits (MMICs) effectively addresses these issues and has achieved significant advancements. The phase shifter chip is a key device in the RF front end. Its phase error and insertion loss (IL) are determined to influence the accuracy of the phase control of each RF channel, and further the quality of the beam steering and scanning. Therefore, it is crucial to design a high-performance phase shifter chip. In the design of a phase shifter, both III-V and silicon technologies are potential candidates with distinct characteristics [1,2,3,4,5,6]. III-V technology offers greater power handling capabilities and lower insertion loss (IL), making it suitable for high-power radar systems. On the other hand, silicon technology boasts lower costs, making it well-suited for commercial applications. The bandwidth and phase shift accuracy of the phase shifter directly affects the performance of these systems. Therefore, the design of broadband-accurate phase shifters has been widely researched in the academic and industrial sectors for decades.

In the literature, many advanced topologies have been studied and developed to achieve a broadband phase shifter. In [1], an improved all-pass network is proposed to extend the relative bandwidth to 40%. In [2], specific logic circuits are added to the phase shifter to simplify the external control circuit. Furthermore, a novel symmetric all-pass network is proposed in [3] to achieve a good reflection coefficient of less than −15 dB at 12–18 GHz. In general, a multi-bit switched phase shifter usually consists of multiple phase shifting cells. Therefore, a large quantity of research on phase shifting cells has been conducted to improve the performance of phase shifters. In [4], various phase shifting cells are proposed to extend the relative bandwidth to 60%, including T-type phase shifting cells, high-pass/low-pass networks, and all-pass networks. In [5], the T-type phase shifting cell is further modified to pursue bandwidth enhancement. In [6], a π-type network is presented to achieve a wideband 90° phase shift with stable performance. With respect to the design of the phase shifting cells, there is significant demand and room for improvement in the 90° phase shifting cells. Related studies focus on replacing the high-pass/low-pass networks with simpler structures to achieve a 90° phase shift in broadband. In practice, significant research has also been conducted to achieve a 90° phase shift with 1-bit phase shifters because of the widespread use of 90° phase shifters. In [7], a 1-bit high-pass/low-pass network is introduced to achieve a 90° phase shift. In [8], a 90° phase shifter is proposed based on MMIC technology. Furthermore, some advanced topologies based on hybrid circuits in [9,10,11,12] are studied and developed to achieve broadband 90° phase shifters. Therefore, designing a broadband and accurate phase shifting cell, particularly a 90° phase shifter, is key to obtaining a broadband phase shifter.

In this paper, a novel 8–18 GHz 90° T-type phase shifter (TPS) is proposed with stable phase shift and superior accuracy. Compared to typical phase shifting cells, especially the conventional T-type phase shifting cells (TPSCs), the proposed TPS integrates a filtering compensation network (FCN) which can efficiently extend the bandwidth. The designed phase shifter is fabricated using 0.15 μm GaAs pHEMT technology. The measured results indicate that the phase error and insertion loss (IL) of the proposed phase shifter are less than ±1.5° and 2.6 dB at 8–18 GHz, respectively. These satisfying performances guarantee the proposed phase shifter is appropriate for ultra-wideband applications.

This paper is organized as follows. In Section 2, an improved TPS is proposed through theoretical analysis of the traditional and typical phase shifting cells, and the performance characteristics of the proposed TPS are verified by extensive simulation experiments. In Section 3, the manufacturing, measurement, and analysis of the proposed TPS are discussed, along with a comparison to the state-of-the-art wideband 90° phase shifters. Section 4 provides a summary of this paper.

## 2. Circuit Design

### 2.1. Typical Switched Phase Shifting Cells

**Figure 1:**
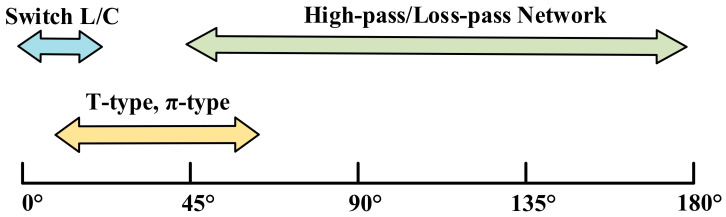
The typical applicability of different phase shifting cells.

Switched L/C type, T-type, π-type, and high-pass/low-pass networks are the most common switched phase shifting cells, and these different topologies have been applied for different phase shifting ranges, as summarized in Figure 1 [1,2,3,4,5,6]. Here the relative phase shift denotes the difference between the phase shifting and the reference states of phase shifting cells. Among these cells, the high-pass/low-pass network offers two individual paths to achieve the reference and phase shift states guaranteeing better isolation and large relative phase shifts. However, the large area required by the two independent paths makes it challenging to meet the demand for compactness. In contrast, the switched L/C type presents a compact area and a simple structure with small relative phase shifts. The conventional T-type structure obtains a medium relative phase shift with medium chip size, while it fails to preserve a stable phase shift within a wide band. Therefore, a promising candidate is to improve the TPSC to enhance its relative phase shift capability with a medium chip area and to preserve stable phase shifts in broadband simultaneously.

### 2.2. Conventional TPSCs

**Figure 2:**
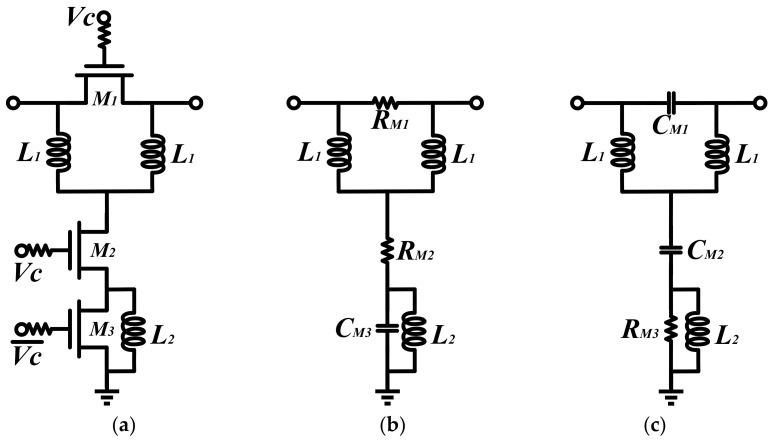
(**a**) The conventional TPSC. (**b**) The equivalent circuit of the reference state and (**c**) the phase shifting state of the conventional TPSC.

As shown in Figure 2, a conventional TPSC consists of three switched transistors controlling the switching of the reference and phase shifting states. The switched transistor can be equivalent to small resistance in on-state and capacitance in off-state. The reference and phase shifting states of the conventional TPSC are shown in Figure 2b and Figure 2c, respectively. Here the $M_{1}$ and the $M_{2}$ are equated to small resistances and the $M_{3}$ is equated to a capacitance in the reference state. The transmission coefficient $S_{21}$ of the reference state can be calculated by the transmission matrix and demonstrated in (1). Correspondingly, in the phase shifting state, the $M_{1}$ and the $M_{2}$ are equated to capacitances, and the $M_{3}$ is equated to a small resistance. The transmission coefficient $S_{21}$ can be calculated in (2) [3,5,6].

(1) $$S_{21,\;\;C,\;r}=\frac{2\omega L_{2}+\omega L_{1}\left(1-\omega^{2}C_{M3}L_{2}\right)}{\omega L_{1}\left(1-\omega^{2}C_{M3}L_{2}\right)-j\left(Z_{0}+\omega L_{2}\left(2j-\omega C_{M3}Z_{0}\right)\right)}$$

(2) $$S_{21,\;\;C,\;p}=\frac{1}{\omega C_{M2}\left(\omega L_{1}-jZ_{0}\right)-2}\times \frac{2Z_{0}\left(1+\omega^{2}C_{M1}L_{1}\left(\omega^{2}C_{M1}L_{1}-2\right)\right)}{\omega L_{1}\left(2\omega C_{M1}Z_{0}-j\right)-Z_{0}}$$

The small equivalent resistances are neglected in (1) and (2) to facilitate the calculation. The insertion phase of the conventional TPSC can be obtained by (1) and (2) as:

(3) $$\varphi_{C,r}={tan}^{-1}\left(\frac{Z_{0}-\omega^{2}C_{M2}L_{2}Z_{0}}{\omega L_{1}+2\omega L_{2}-\omega^{3}C_{M3}L_{1}L_{2}}\right)$$

(4) $$\varphi_{C,p}={-tan}^{-1}\left(\frac{\omega (2L_{1}+C_{M2}Z_{0}^{2}-\omega^{2}C_{M2}L_{1}^{2}-2\omega^{2}C_{M1}C_{M2})}{2Z_{0}(1-2\omega^{2}C_{M1}L_{1}-\omega^{2}C_{M2}L_{1}+\omega^{4}C_{M1}C_{M2}L_{1}^{2}}\right)$$

Therefore, the relative phase shift $\Delta \varphi_{C}$ can be acquired by the insertion phase difference between the two states as follows:

(5) $$\Delta \varphi_{C}=\varphi_{C,r}-\varphi_{C,p}$$

From (5), it can be seen that the conventional TPSC exhibits significant unevenness in broadband. And the parasitic effects of the transistors at high frequencies appear more sensitive, resulting in the deterioration of phase shift flatness. Therefore, the conventional TPSC needs to be modified to accommodate higher frequency and wider bandwidth systems.

### 2.3. Improved TPSCs

**Figure 3:**
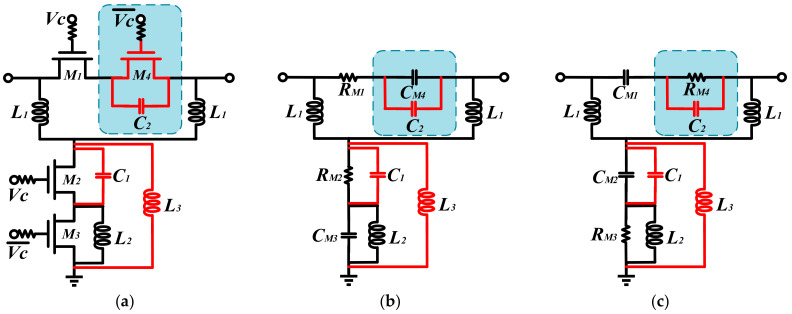
(**a**) The improved TPSC. (**b**) The equivalent circuit of the reference state and (**c**) the phase shifting state of the improved TPSC.

**Figure 4:**
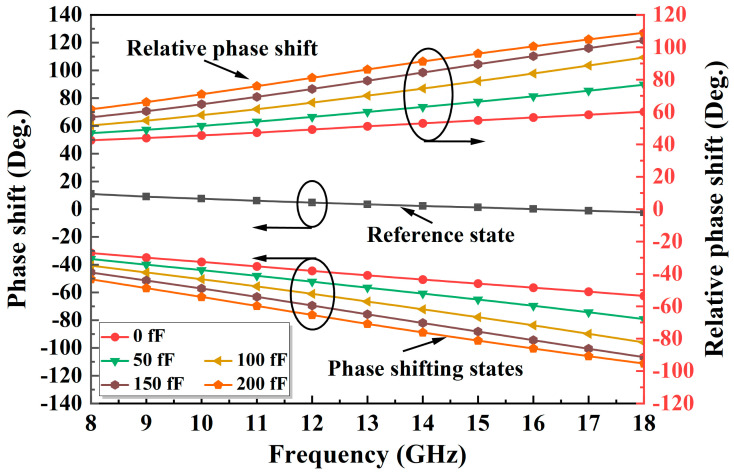
The phase shift improvements with different $C_{1}$ values.

**Figure 5:**
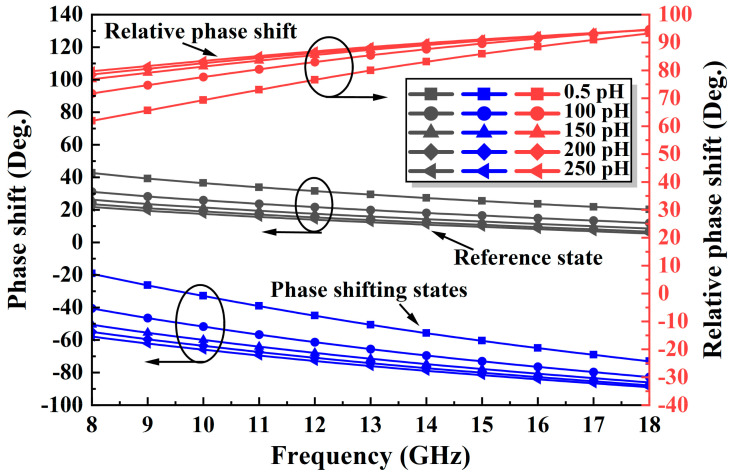
The phase shift improvements with different $L_{3}$ values.

**Figure 6:**
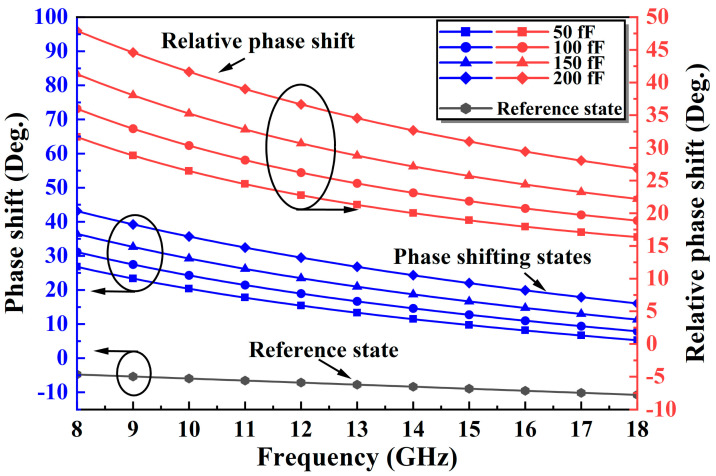
The phase shift variation with different $C_{2}$ values.

**Figure 7:**
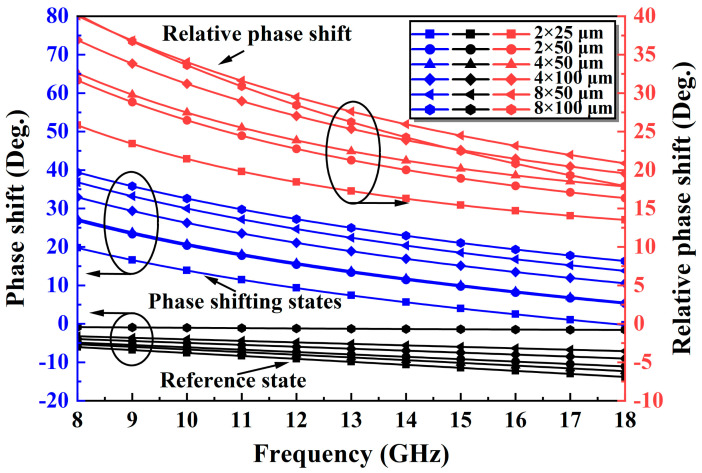
The phase shift variation with different $M_{4}$ values.

An improved TPSC is designed to address the issue of unstable phase shift over a wide band in a conventional TPSC. As shown in Figure 3a, the improved TPSC adds a capacitance $C_{1}$ in parallel with the $M_{2}$, an inductance $L_{3}$ in parallel with the $M_{2}$ and the $M_{3}$, and an FCN consisting of the $M_{4}$ and the $C_{2}$ in series. The $C_{1}$ can compensate for the insufficient phase shifting capacity caused by the inadequate capacitance value of the $M_{2}$, thus achieving a breakthrough in phase shift. As shown in Figure 4, the reference state is expected to remain constant as the $C_{1}$ increases, while the insertion phase of the phase shifting state keeps increasing in the meantime. Therefore, the introduction of the $C_{1}$ can effectively increase the phase shift capability of the TPSC. Furthermore, the added shunt inductor $L_{3}$ can effectively solve the problem of broadband phase shifting flatness. Figure 5 shows the phase change of the TPSC for different $L_{3}$ values. With an increment of the $L_{3}$, both the phase shifting capability and the relative phase flatness are significantly improved. To further improve the phase flatness of the TPSC, an FCN is employed, as shown in Figure 3a. In theory, the FCN can also be equated to a miniature phase shifter. When the control voltages of the $M_{4}$ and the $M_{1}$ are opposite, the FCN and the TPSC provide relative phase shifts in the same direction, but with opposite trends in frequency. As shown in Figure 6 and Figure 7, the FCN exhibits different phase shift degrees for different $C_{2}$ values and different $M_{4}$ sizes. Therefore, the addition of the FCN can effectively enhance the phase shifting capacity and appropriate $M_{4}$ and $C_{2}$ can significantly improve the phase flatness of the TPSC. In general, the reasonable selection of suitable values of the $C_{1}$, the $L_{3}$, the $C_{2},$ and the $M_{4}$ can guarantee strong phase shifting capability and good phase flatness of the TPSC.

The reference state and phase shifting state of the improved TPSC are shown in Figure 3b,c. In the reference state, $M_{1}$ and $M_{2}$ are turned on, and $M_{3}$ and $M_{4}$ are turned off. The $S_{21}$ of the reference and phase shifting states can also be calculated from the transfer matrix as shown in (6) and (7).

(6) $$S_{21,I,r}=\frac{1}{Z_{0}+\omega L_{1}\left(j-2\omega \left(C_{2}+C_{M4}\right)Z_{0}\right)}\times \frac{2\omega \left(L_{2}L_{3}-\omega^{2}{L_{1}L}_{3}\left(C_{2}+C_{M4}\right)\left(2L_{2}+L_{1}-\omega^{2}C_{M3}L_{2}L_{1}\right)-\omega^{2}L_{1}^{2}L_{2}\left(C_{2}+C_{M4}\right)\right)Z_{0}}{\omega L_{1}\left(1-\omega^{2}\left(C_{1}+C_{M2}\right)L_{3}\right)-jZ_{0}+\omega L_{3}\left(2+j\omega \left(C_{1}+C_{M2}\right)Z_{0}\right)}$$

(7) $$S_{21,I,p}=\frac{1}{Z_{0}+\omega L_{1}\left(j-2\omega C_{M1}Z_{0}\right)}\times \frac{2Z_{0}\omega^{3}C_{M1}L_{1}\left(2L_{3}+L_{1}-\omega^{2}L_{3}L_{1}\left(C_{1}+C_{M2}\right)\right)-2Z_{0}\omega L_{3}}{\omega^{3}L_{3}L_{1}\left(C_{1}+C_{M2}\right)-\omega L_{1}-2\omega L_{3}+jZ_{0}-jZ_{0}\omega^{2}L_{3}\left(C_{1}+C_{M2}\right)}$$

In (6) and (7), the small equivalent resistance is ignored to facilitate the calculation, and the corresponding insertion phases are shown in (8) and (9), respectively.

(8) $$\varphi_{I,r}=\frac{\omega^{2}L_{1}^{2}\left(L_{3}+L_{2}-\omega^{2}C_{M3}L_{3}L_{2}\right)-Z_{0}^{2}\left(L_{3}+L_{2}-\omega^{2}C_{M3}L_{3}L_{2}\right)+2\omega^{2}L_{1}\left(L_{2}L_{3}+Z_{0}^{2}\left(C_{2}+C_{M4}\right)\left(L_{2}+L_{3}\right)\right)}{2\omega Z_{0}\left(L_{2}L_{3}-\omega^{2}L_{1}^{2}\left(C_{2}+C_{M4}\right)\left(L_{2}+L_{3}\right)+L_{3}L_{1}+L_{2}L_{1}-\omega^{2}L_{3}L_{2}L_{1}\left(2\left(C_{2}+C_{M4}\right)+C_{M3}\right)\right)\;}$$

(9) $$\varphi_{I,p}=\frac{\omega^{2}L_{1}\left(2L_{3}+L_{1}-\omega^{2}L_{3}L_{1}\left(C_{1}+C_{M2}\right)\right)-Z_{0}^{2}+{Z_{0}^{2}\omega }^{2}\left(2C_{M1}L_{1}+L_{3}\left(C_{1}+C_{M2}\right)\right)}{2Z_{0}\omega {(L}_{3}+L_{1})-2Z_{0}\omega^{3}L_{1}(C_{M1}L_{1}+L_{3}(C_{1}+2C_{M1}+C_{M2}))}$$

The relative phase shift of the improved TPSC can be obtained by (8) and (9) as follows:

(10) $$\Delta \varphi_{I}=\varphi_{I,r}-\varphi_{I,p}$$

**Figure 8:**
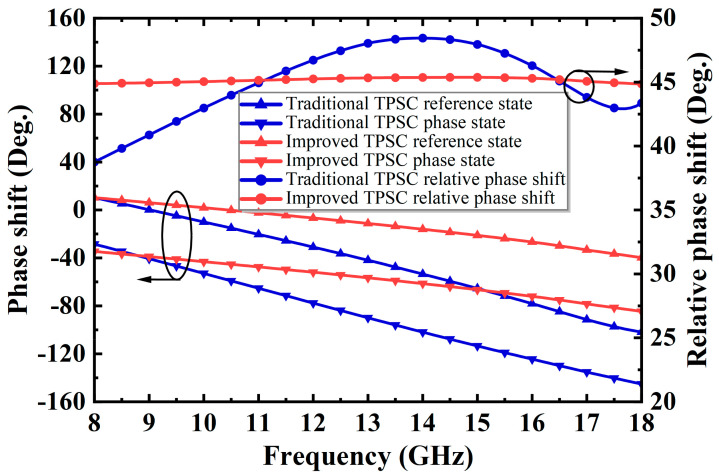
The performance comparison of the traditional and the improved TPSC at 45° phase shift.

**Figure 9:**
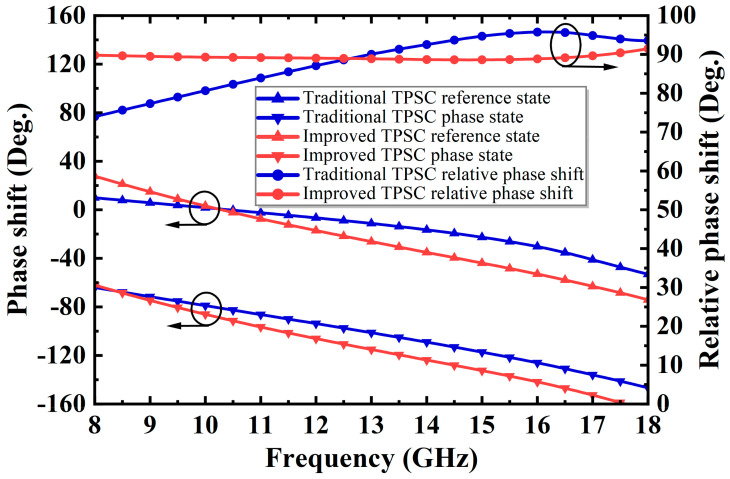
The performance comparison of the traditional and the improved TPSC at 90° phase shift.

Compared to (5), it can be seen in (10) that the proposed TPSC exhibits stronger phase shift capability and better phase flatness over a wide band than traditional TPSC. Figure 8 and Figure 9 further illustrate this conclusion. As shown in Figure 8, when the traditional and the improved TPSC both operate within a lower phase shift range, the improved one exhibits a flatter phase shift compared to the traditional one. As shown in Figure 9, when the traditional TPSC is forced to a 90° phase shift, it becomes unusable over a wide frequency band. On the contrary, the improved TPSC can achieve a flat 90° phase shift in the desired band.

## 3. Measured Results

**Figure 10:**
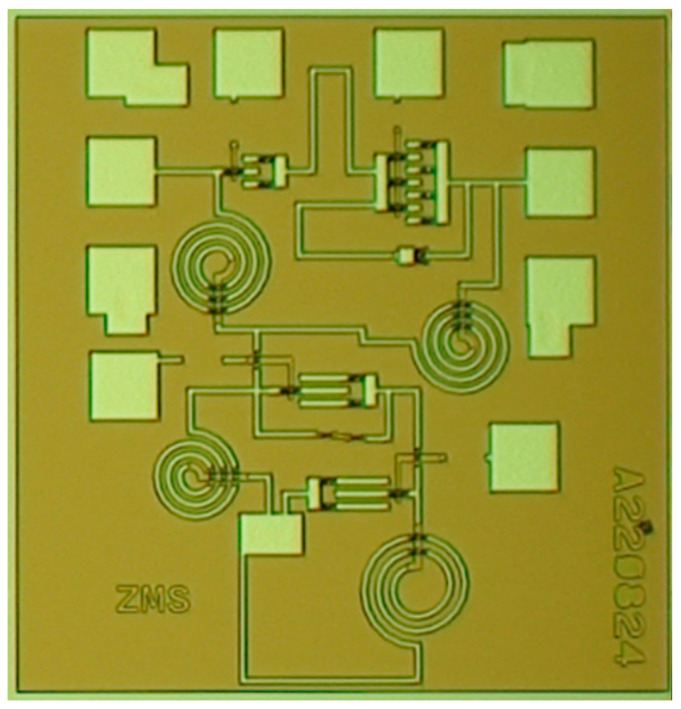
The micrograph of the improved TPS.

**Figure 11:**
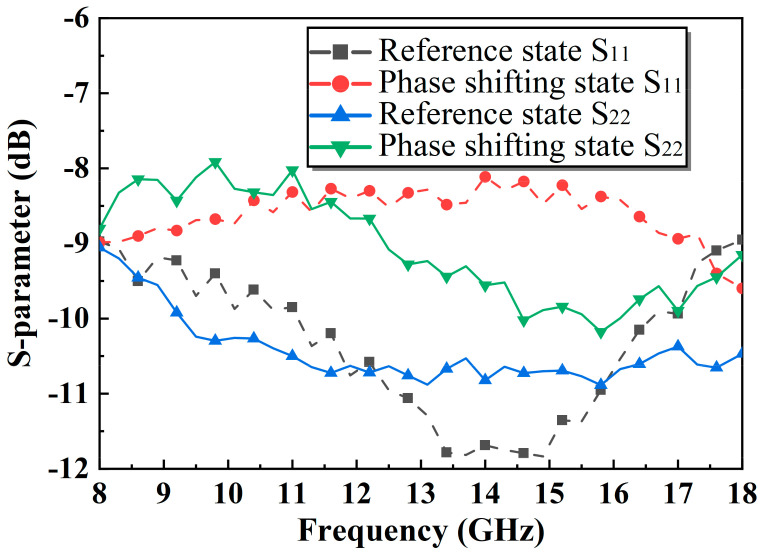
The measured input and output reflection coefficient.

**Figure 12:**
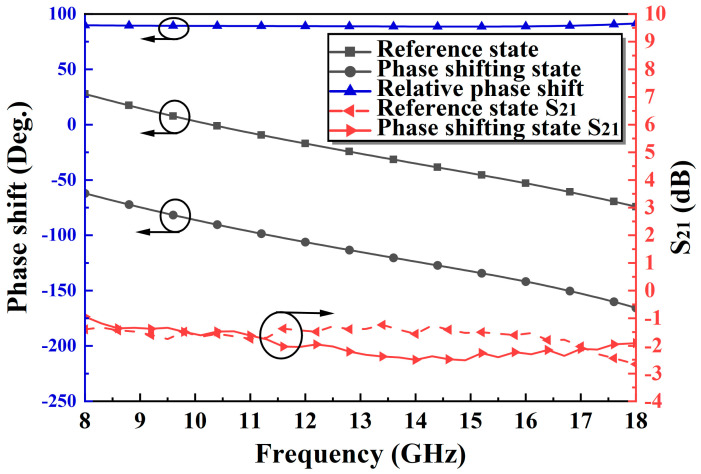
The measured IL and insertion phases of two states, and the measured relative phase shift.

Based on the above analysis, a novel 90° TPS is fabricated using 0.15-μm GaAs pHEMT technology, and the micrographs are shown in Figure 10 with a small area of merely 0.53 × 0.86 mm^2^. The main parameters of the proposed TPS are presented in Table 1. The proposed phase shifter is measured on-wafer using a probe station (Cascade Summit 11000M, Beaverton, OR, USA), and a vector network analyzer (Agilent N5244A, Santa Clara, CA, USA). The measured $S_{11}$ and $S_{22}$ are both less than −8 dB at 8–18 GHz, as displayed in Figure 11. The proposed phase shifter exhibits a small IL of 1.3–2.6 dB, and a small phase error of merely ±1.5 dB at 8–18 GHz, as shown in Figure 12. Table 2 summarizes the proposed improved TPS with the state-of-the-art wideband 90° phase shifters. These excellent performance characteristics demonstrate that the proposed phase shifter is well-suited for various ultra-wideband systems.

## 4. Conclusions

This paper proposes a novel improved TPS which achieves a 90° phase shift at 8–18 GHz using 0.15-μm GaAs pHEMT technology. A TPS with integrated FCN is proposed by theoretical analysis of typical phase shifting cells, especially the conventional TPSC. The proposed TPS breaks the relative phase shift limitation of the traditional TPSC and preserves the stable phase shift. The measurements indicate the fabricated structure has only ±1.5° phase error in the range of 8–18 GHz at a 90° phase shift with good impedance matching of $S_{11}$ and $S_{22}$ less than −5 dB. Therefore, the designed phase shifter has good performance and is suitable for wideband wireless and radar systems.

## Figures and Tables

**Table 1:** The main parameters of the proposed TPS.

$M_{1}$	$M_{2}$	$M_{3}$	$M_{4}$	
2×30 μm	2×75 μm	2×80 μm	6×25 μm	
$C_{1}$	$C_{2}$	$L_{1}$	$L_{2}$	$L_{3}$
13 fF	190 fF	1.1 pH	0.85 pH	0.68 pH

**Table 2:** Summary and comparison of the state-of-the-art wideband 90° phase shifters.

Reference	BW (GHz)	BW (%)	Phase Shift (°)	Error (°)	$S_{11}$$,\;S_{22}$ (dB)	IL (dB)	Process
[7] MWCL’2015	57–63	10	90	±3	<−10, <−10	<5.2	65 nm CMOS
[8] MWCL’2009	50–65	26	90	±1	<−11, <−11	<8	90 nm CMOS
[13] MWCL’2019	5–6	18.2	90	±2	<−11, <−11	<4 *	0.18 μm CMOS
[14] TMTT’2010	S/C	10	90	±3	<−10, <−10	<7 *	0.5 μm pHEMT
[15] TMTT’2010	2.5–3.2	25	90	±2	<−8, <−9	<3 *	0.18 μm CMOS
[4] MWCL’2020	3–6	66.7	90	±2	<−8, <−8	<6	0.5 μm pHEMT
**This work**	**8–18**	**77**	**90**	**±1.5**	**<−8, <−8**	**<2.6**	**0.15 μm pHEMT**

*: The estimated IL of the 90° phase shifter cell from the figure of the measured results.

## Data Availability

The data that support the findings of this study are available from the corresponding author upon reasonable request.

## References

[1] Bahl I.J., Conway D. (2008). L- and S-Band Compact Octave Bandwidth 4-bit MMIC Phase Shifters. IEEE Trans. Microw. Theory Tech..

[2] An J., Wang G.-M., Zhang C.-X., Qu S.-B., Zhang P. (2008). Broadband digital phase shifter based on composite right/left-handed transmission line. Microw. Opt. Technol. Lett..

[3] Zeng J., Xie C., Zhang D., Feng J., Tan C., Yu Z. (2023). A wideband accurate compact 6-bit phase shifter in 0.15-μm GaAs technology. Microw. Opt. Technol. Lett..

[4] Chen J., Mou S., Ma K., Meng F. (2020). A 3–6-GHz Wideband Compact 6-Bit Phase Shifter in 0.5-μm GaAs Technology. IEEE Microw. Wirel. Compon. Lett..

[5] Li X., Fu H., Ma K., Hu J. (2022). A 2.4–4-GHz Wideband 7-Bit Phase Shifter with Low RMS Phase/Amplitude Error in 0.5-μm GaAs Technology. IEEE Trans. Microw. Theory Tech..

[6] Kang D.W., Lee H.D., Kim C.H., Hong S. (2006). Ku-band MMIC phase shifter using a parallel resonator with 0.18-μm CMOS technology. IEEE Trans. Microw. Theory Tech..

[7] Song I.S., Yoon G., Park C.S. (2015). A Highly Integrated 1-Bit Phase Shifter Based on High-Pass/Low-Pass Structure. IEEE Microw. Wirel. Compon. Lett..

[8] Biglarbegian B., Nezhad-Ahmadi M.R., Fakharzadeh M., Safavi-Naeini S. (2009). Millimeter-Wave Reflective-Type Phase Shifter in CMOS Technology. IEEE Microw. Wirel. Compon. Lett..

[9] Lyu Y.P., Zhu L., Cheng C.H. (2019). A New Design of Ultrawideband Single-Layer 90° Phase Shifter in the View of Group Delay. IEEE Microw. Wirel. Compon. Lett..

[10] Honari M.M., Mirzavand R., Mousavi P. (2018). Wideband and Ultrawideband Phase Shifter Designs Based on Low-Pass/Bandpass/High-Pass Networks. IEEE Trans. Compon. Packag. Manuf. Technol..

[11] Zhang W., Xu K., Shi J., Shen Z. (2020). A Compact Single-Layer Balanced Phase Shifter with Wide Bandwidth and Uniform Reference Line. IEEE Access.

[12] Dong Q., Wu Y., Zheng Y., Wang W., Liu Y. (2019). A Compact Single-Layer Ultra-Wideband Phase Shifter Using Weakly Coupled Lines. IEEE Access.

[13] Jeon H., Kobayashi K.W. (2019). A High Linearity +44.5-dBm IP3 C-Band 6-Bit Digital Phase Shifter Using SOI Technology for Phased Array Applications. IEEE Microw. Wirel. Compon. Lett..

[14] Hangai M., Hieda M., Yunoue N., Sasaki Y., Miyazaki M. (2010). S- and C-Band Ultra-Compact Phase Shifters Based on All-Pass Networks. IEEE Trans. Microw. Theory Tech..

[15] Meghdadi M., Azizi M., Kiani M., Medi A., Atarodi M. (2010). A 6-Bit CMOS Phase Shifter for S-Band. IEEE Trans. Microw. Theory Tech..

